# ZmDof30 Negatively Regulates the Promoter Activity of the Pollen-Specific Gene *Zm908*

**DOI:** 10.3389/fpls.2017.00685

**Published:** 2017-05-01

**Authors:** Jing Peng, Xin Qi, Xiyang Chen, Nan Li, Jingjuan Yu

**Affiliations:** State Key Laboratory of Agrobiotechnology, College of Biological Sciences, China Agricultural UniversityBeijing, China

**Keywords:** maize, *Zm908*, pollen-specific, *cis*-regulatory elements, ZmDof30, transcriptional repressor

## Abstract

The maize (*Zea mays*) pollen-predominant gene *Zm908*, a novel small-peptide gene, was reported to play critical roles in pollen germination and pollen tube growth in our previous work. In this study, we aimed to explore the regulatory mechanism of *Zm908*. The putative promoter of *Zm908* was cloned and analyzed. The activity analysis of a series of promoter truncations in different tissues of transgenic tobacco plants indicated that the *Zm908* promoter is pollen-specific and that the –126 to –68 region is crucial for pollen expression. The 5′ deletion analysis of the –126 to –68 region revealed that the –126 to –102 region functions as a transcriptional suppression element. *ZmDof30*, which is predominantly expressed in pollen and whole anthers, was cloned and characterized. ZmDof30-GFP localized to the nuclei of maize protoplasts and possessed no transcriptional activation activity in a yeast system. ZmDof30 could bind to the AAAG elements in p184 sequence containing the –126 to +58 region of the *Zm908* promoter *in vitro* and *in vivo*, and negatively regulated p184 activity in tobacco leaves. Collectively, ZmDof30 may function as a *Zm908* transcriptional repressor in pollen, and these results may provide a better understanding of the regulation of the *Zm908* gene. Additionally, the pollen-specific *Zm908* promoter may be valuable for genetically engineering male sterility.

## Introduction

In recent years, a novel class of small-peptide genes has been identified in plants. These genes contain one or more short open reading frames (sORFs), encoding small peptides with less than 100 amino acid residues (Kastenmayer et al., 2006). Many plant small peptides encoded by sORFs are involved in plant morphogenesis processes. In *Arabidopsis thaliana*, the INFLORESCENCE DEFICIENT IN ABSCISSION (IDA) gene encodes a small peptide protein that controls floral organ abscission (Butenko et al., 2003). The overexpression of *Zm401* (AY911609), an anther-specific sORF gene in maize, leads to pollen sterility in transgenic tobacco and maize (Dai et al., 2007; Ma et al., 2008). *Zm401p10*, the longest variant of *Zm401*, plays significant roles in tapetum and microspore development (Wang et al., 2009). Further analysis of the regulation of *Zm401* expression showed that Dof1 could down-regulate *Zm401* expression in transgenic tobacco pollen by interacting with the *Zm401* upstream promoter region (–670 to –510) which contains tandem Dof common recognition elements (Chen et al., 2012). Dong et al. (2013) identified a pollen-predominant sORF gene, *Zm908*, which is homologous to *Zm401*. The longest *Zm908* sORF encodes a predicted protein, Zm908p11, with 97 amino acids. The *Zm908* transcript was first detected at low levels in the uninucleate stage of the anthers and reached maximum expression in mature pollen. Additionally, only weak expression of *Zm908* was detected in leaves, and no expression was detected in other tissues. GFP fusion protein localization assays revealed that Zm908p11 was distributed in the pollen tube. *Zm908p11* overexpression dramatically decreased pollen germination efficiency in maize and tobacco. Zm908p11 could also interact with profilin to prevent profilin-actin nucleation, which is involved in the regulation of pollen tube growth (Dong et al., 2013).

Multiple Dof family members have been identified in numerous evolutionarily distant plants, both monocots and dicots, including maize (Vicente-Carbajosa et al., 1997; Yanagisawa and Sheen, 1998), barley (Mena et al., 1998), tobacco (Cai et al., 2013; Corrales et al., 2014) and *Arabidopsis* (Wei et al., 2010). Dof transcription factors contain a strikingly conserved DNA-binding domain that located close to the N-terminal region. This domain can specifically bind to cis-regulatory elements. The cis-regulatory elements contain a common recognition core, 5′-(T/A)AAAG-3′, which was named as Dof core element (Yanagisawa and Schmidt, 1999; Yanagisawa, 2002). The Dof core element is unique and a single-base mutation can abolish Dof transcription factor binding to DNA, particularly the A at position +2 and the G at position +4 (Yanagisawa and Sheen, 1998; Yanagisawa and Schmidt, 1999). The sequences flanking the core recognition element also influence the DNA-binding activity of Dof transcription factors, but the effects are not as dramatic as those following mutation of the core sequence (Yanagisawa and Schmidt, 1999). Dof transcription factors have variable C-terminal domains. These regions function in transcriptional regulation, mediating activation or repression of gene expression by interacting with other regulatory proteins, and are likely to contribute to the diverse functions of Dof transcription factors. The Dof members identified thus far participate in the regulation of genes involved in diverse physiological processes, such as plant hormone responses (Kisu et al., 1997; Baumann et al., 1999; Mena et al., 2002), photosynthesis (Yanagisawa, 2000; Gupta et al., 2014), stress responses (Cai et al., 2016), seed germination (Santopolo et al., 2015), stomatal maturation and functioning (Negi et al., 2013), endosperm development (Vicente-Carbajosa et al., 1997; Marzabal et al., 2008; Qi et al., 2016) and pollen development (Chen et al., 2012).

In this study, we cloned and analyzed the putative promoter of *Zm908* (pZm908). Through an activity analysis of a series of promoter truncations in different tissues of transformed tobacco, the expression of pZm908 was investigated. The full-length of pZm908 was pollen-specific and the –126 to –68 region was crucial for promoter activity. The 5′ deletion analysis of the –126 to –68 region showed that the –126 to –102 region of pZm908 acted as a negative element. Deletion of the –126 to –102 region resulted in lacking one Dof core element, destruction of two tandemly repeated Dof core elements and lacking one lat52 pollen-specific transcription activation cis-regulatory elements indicating that the Dof core elements were likely negative regulatory elements and a Dof transcription factor might participate in suppressing the promoter activity. ZmDof30, a pollen-predominant Dof transcription factor, interacted directly with the AAAG motif in –126 to –100 region of the *Zm908* promoter *in vitro* and *in vivo*, and negatively regulated the activity of p184 containing the –126 to +58 region of *Zm908* promoter in tobacco leaves. Collectively, ZmDof30 may function as a *Zm908* transcriptional repressor in pollen, and these results provide a better understanding of the regulation of *Zm908* in pollen germination and pollen tube growth.

## Materials and Methods

### Construction of Vectors Containing Truncated pZm908 Fused with a β-Glucuronidase (GUS) Reporter

pZm908 was cloned from the key maize inbred line, Zong31, which was cultivated by the Chinese scientist Jingrui Dai (China Agricultural University), and fused with the GUS gene in the pBI121 plasmid to generate p908::GUS. For the generation of the truncated constructs, p908::GUS was used as a template for PCR using 2× Taq Platinum PCR Master Mix (TIANGEN, China). Primers designed with restriction endonuclease sites are listed in Supplementary Table S1. The PCR fragments were then cloned into the pMD^TM^19-T simple vector (TaKaRa) for sequencing. The inserts were digested from the recombinant pMD^TM^19-T simple vector and cloned into the corresponding restriction sites of pBI121. Each recombinant vector was confirmed by digestion and sequencing.

### Tobacco Transformation and Selection

Sterile tobacco seedlings (*Nicotiana tabacum* cv. Xanthi-nc) grown in bottles with Murashige and Skoog (MS) medium at 26°C under a 16 h light/8 h dark photoperiod were used to obtain stably transformed lines in this study. Each truncated construct was introduced into tobacco by *Agrobacterium tumefaciens* (LBA4404)-mediated leaf disk transformation (Gallois and Marinho, 1995). After selection on kanamycin-containing medium, transgenic tobacco lines were identified by PCR and then grown in the greenhouse.

### GUS Histochemical Staining Assay

The leaves and roots of tobacco seedlings, immature seeds, indehiscent anthers and mature pollen shed from intraday dehiscent anthers were used in the GUS histochemical staining assay. GUS staining was performed using the chromogenic substrate 5-bromo-4-chloro-3-indolyl-β-D-glucuronic acid (X-gluc; Bio Vectra, Oxford, CT, USA). Each sample was incubated with GUS staining buffer (1 mM X-gluc solution in 0.5 M EDTA, 5 mM FeK_3_(CN)_6_, 5 mM K_4_Fe(CN)_6_, 100 mM sodium phosphate buffer [pH 7.0] and 0.1% Triton X-100) for 12 h at 37°C. Following alcohol decolorization, the samples were photographed under a microscope (Olympus SEX16, Tokyo, Japan).

### Fluorometric Quantitative Analysis of GUS Activity

Total protein was extracted from mature pollen grains using extraction buffer (50 mM sodium phosphate (pH 7.0), 10 mM EDTA, 0.1% SDS, 10 mM β-mercaptoethanol, and 0.1% Triton X-100). After vortexing, the samples were incubated on ice for 15 min and then centrifuged at 13,500 *g* for 10 min at 4°C. The supernatant containing the extracted protein was collected and stored at 4°C. The protein concentration was determined as described by Bradford (Bradford, 1976). The fluorometric measurement of GUS activity was performed using 4-methylumbelliferone (4-MU) β-D-glucuronide hydrate (MUG; Sigma, St. Louis, MO, USA) as the substrate and employing a F-4500 fluorescence spectrophotometer (Hitachi, Tokyo, Japan) to measure fluorescence. GUS activity, expressed as nanomoles of 4-MU produced per gram protein per minute, was normalized to total protein concentration.

### Subcellular Localization

The *ZmDof30* coding region, cloned from maize anther cDNA, was fused to GFP in the pUC-GFP plasmid to construct the ZmDof30-GFP vector. The AT-hook motif nuclear-localized protein 22 gene (*AHL22*) was used as a positive control. For subcellular localization assays, maize protoplasts were isolated according to a previously reported protocol (Yoo et al., 2007) and were co-transfected with AHL22-RFP and ZmDof30-GFP. GFP was used as negative control. The transfected protoplasts were incubated 12–16 h at 28°C in the dark. Fluorescence was detected using a confocal laser scanning microscope (ZEISS LSM 510).

### Transcriptional Activation Assay in Yeast

The yeast strain YRG-2, containing *HIS3* and *lacZ* reporter genes, was used as an assay system (Stratagene, USA). The *ZmDof30* coding region fused with GAL4 was inserted into the pBD-GAL4 plasmid using the *Eco*RI and *Sal*I sites to generate the pBD-ZmDof30 vector. The pBD-ZmDof30, pBD-GAL4 (negative control), and pGAL4 (positive control) plasmids were transfected into yeast. The transfected yeast cells were cultured on YPDA or SD/-Trp/-His medium (lacking threonine and histidine) at 30°C for 3 days. β-galactosidase activity assays of the transfected yeast cells were performed as described in the Yeast Protocols Handbook (Clontech, PT3024-1).

### Yeast One-Hybrid

The bait sequence double E (p184), synthesized in tandem, was cloned into pAbAi to generate the bait plasmid (**Figure 4A**), which was transformed into yeast strain Y1HGold according to the instructions of the Yeastmaker^TM^ Yeast Transformation System 2 (Clontech, USA). As a negative control, the E (p184)-M sequence was mutated from AAAG to AATC. Bait strains were selected from colonies that grew well on a synthetic defined (SD) medium lacking uracil. The coding sequence of *ZmDof30* was cloned into the *Eco*RI and *Bam*HI sites of pGADT7 to generate the prey vector. After screening for the minimal inhibitory concentration of aureobasidin A (AbA), the bait strains were transformed with the prey vector and cultured on SD medium lacking leucine and containing the minimal inhibitory concentration of AbA at 30°C for 3 days.

### EMSA

Full-length ZmDof30 could not be expressed in the prokaryotic expression system. Therefore, the truncated fragment encoding the 26–127 amino acids, which contains the DNA binding domain of ZmDof30, was cloned into pMAL-C2x to construct the MBP-ZmDof30_26-127aa_ vector. The MBP-ZmDof30_26-127aa_ fusion protein was expressed in TB1 and purified using affinity chromatography. The oligonucleotide probes were synthesized and labeled with biotin. Double stranded probes can be annealed at 30°C overnight after PCR denaturation at 95°C for 5 min. A gel-shift assay was performed using the LightShift^®^ Chemiluminescent EMSA kit (Prod # 89880, Thermo, USA) according to the manufacturer’s instructions.

### Transient Expression Assay in *Nicotiana benthamiana*

*Nicotiana benthamiana* plants at the six-leaf stage, grown in a phytotron at 23°C with a 16 h light/8 h dark cycle, were used for transient transformation. The reporter plasmid (p184::GUS), the effector plasmid (pSuper::ZmDof30) and the internal control (35S::LUC) were transformed into *Agrobacterium* (GV3101) individually and then co-infiltrated into the leaves of *N. benthamiana* as previously described (Chen et al., 2009). The empty pSuper vector was used as a negative control. Three days after inoculation, GUS and luciferase (LUC) activities in protein extracts prepared from the infiltrated leaves were monitored, and the relative GUS/LUC activity was used for the quantitation of promoter activity. The quantification of LUC activity was performed in accordance with the manufacturer’s protocol (Promega).

## Results

### Isolation and Analysis of the *Zm908* Putative Promoter

The putative promoter sequence of *Zm908*, designated here as pZm908, was cloned from Zong31. It was 2,126 bp in length, comprising 2,068 nucleotides immediately upstream from the putative transcription start site of the *Zm908* gene and 58 nucleotides of the 5′ untranslated region of the *Zm908* gene. Bioinformatics analysis of pZm908 identified several pollen-specific cis-regulatory elements, including the AGAAA motif, the GTGA motif and the Q-element (**Table 1**). AGAAA is a pollen-specific transcription activation cis-regulatory element of the tomato *lat52* gene (Bate and Twell, 1998). The GTGA motif, conserved in the promoter of the tobacco late pollen gene *g10* and the tomato pollen gene *lat56*, participates in directing pollen-specific expression (Rogers et al., 2001). The Q-element can enhance the expression of the pollen-specific maize gene *ZM13*, whereas it shows no independent activity in pollen (Hamilton et al., 1998). These putative pollen-specific cis-regulatory elements in pZm908 may help drive the pollen-predominant expression of *Zm908*.

**Table 1:** Putative pollen-specific *cis*-elements in pZm908.

*Cis*-element	Detected in the Zm908 promoter sequence	Motif position	Function	Reference
POLLEN1LELAT52	AGAAA	–102 to –97	A pollen-specific transcription activation	Bate and Twell, 1998
		–108 to –103	*cis*-regulatory element of the tomato *lat52* gene	
		–866 to –861		
		–1916 to –1911		
GTGA motif	GTGA	–194 to –190	Conserved in the promoter of the tobacco late pollen	Rogers et al., 2001
		–395 to –391	gene *g10* and tomato pollen gene *lat56*	
		–625 to –621		
		–757 to –753		
		–1458 to –1454		
POLLEN Q-element	AAATGA	–97 to –91	Enhances the expression of the pollen-specific maize gene, *ZM13*	Hamilton et al., 1998
TATA BOX	TCTATAAATT	–154 to –144	Control the level of transcription and determines the location and number of transcription start sites	Joshi, 1987
CAAT-BOX	CCAATT	–137 to –131	A common cis-acting element in promoter and enhancer regions	Delvoye et al., 1993

### Construction and Analysis of Truncation Series for pZm908

**FIGURE 1:**
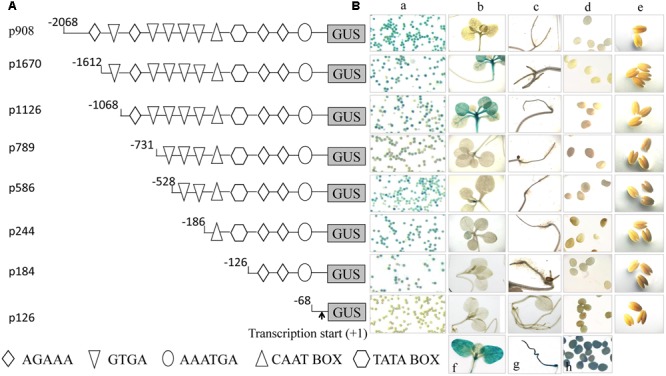
**Analysis of pZm908 deletion mutants in transgenic tobacco plants. (A)** Deletion constructs consisting of putative pollen-specific cis-regulatory elements as shown in **Table 1**. **(B)** Histochemical staining of GUS activity in the transgenic tobacco of pZm908 deletion constructs **(a–e)**. **(B-a)** The mature pollen grain, **(B-b)** seedling leaves, **(B-c)** seedling roots, **(B-d)** immature seeds, **(B-e)** indehiscent anthers, **(B-f)** leaves of 35S::GUS transgenic tobacco as a positive control, **(B-g)** root of 35S::GUS transgenic tobacco as a positive control, and **(B-h)** immature seeds of p19Z::GUS transgenic tobacco as a positive control. Mature pollen grains were collected from intraday dehiscent anthers. Three independently isolated transgenic tobacco lines were analyzed for p1670, p1126, p789, p184, and p126; two lines were analyzed for p908, p586 and p244.

To identify functional regions in pZm908, a promoter deletion series (p908, p1670, p1126, p789, p586, p244, p184, and p126) was prepared as outlined in **Figure 1A** and used to generate GUS reporter gene constructs. These constructs were analyzed using a tobacco system due to the sufficient quantity of pollen grains and the high transformation efficiency of this system, which enabled many promoter variants to be evaluated in detail as stably transformed lines. We performed GUS staining in the leaves and roots of seedlings, mature pollen grains, indehiscent anthers and immature seeds collected from the transgenic plants. The results showed that no apparent GUS activity was observed following staining with X-gluc in seedling roots, immature seeds or indehiscent anthers for any construct (**Figure 1**). p1670 and p1126 showed strong GUS staining in seedling leaves. GUS staining was detected in mature pollen with all constructs except p126. GUS staining was found in both mature pollen grains and leaves of seedlings for p1670 and p1126, consistent with the *Zm908* gene expression pattern in maize (Dong et al., 2013). The full-length pZm908, p908, showed high GUS staining intensity in mature pollen grains, whereas no apparent GUS staining was detected in the other four tissues, indicating that p908 was pollen-specific. The p126 deletion constructs, which were missing the –126 to –68 region from p184, showed no GUS staining in mature pollen grains, indicating that the –126 to –68 region was crucial for pollen expression.

### The AAAG Motif in p184 Is a Transcription Suppression Element

**FIGURE 2:**
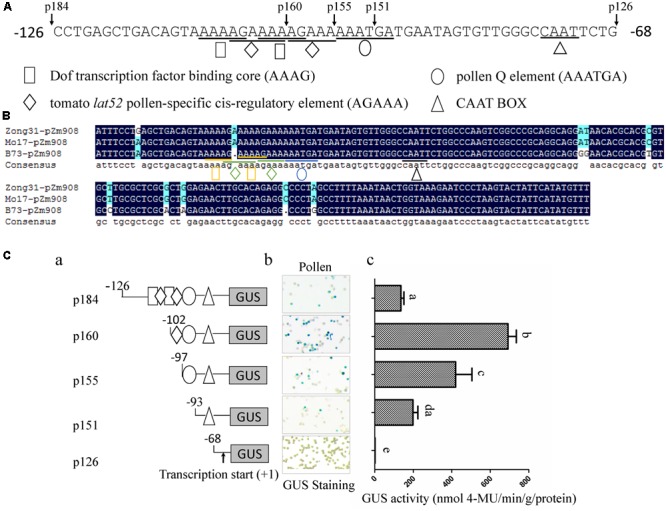
**Analysis of p184 promoter deletion mutants in transgenic tobacco plants. (A)** The sequence of –126 to –68 region of pZm908 in Zong31. The location of the deletion constructs and putative cis-regulatory elements are indicated. **(B)** Homology analysis of p184 segments in Zong31, Mo17 and B73. The putative cis-regulatory elements are indicated. **(C-a)** Deletion constructs consisting of putative cis-regulatory elements located in p184. **(C-b)** Histochemical staining of GUS activity in mature pollen grains of transgenic tobacco plants. **(C-c)** Scatter histograms showing quantitative determination of GUS activity in mature pollen grains of transgenic tobacco plants. Four individual lines harboring each p184 promoter deletion construct were analyzed. The values represent the mean ± SD from four separate plants of each construct. Significant differences were assessed by the Student’s *t*-test using a *P*-value < 0.05.

An analysis of *cis*-acting elements revealed that there were two tandem repeated Dof core elements (AAAG), two *lat52* pollen-specific cis-regulatory elements (AGAAA), one pollen Q element and one CAAT box in the –126 to –68 region. One of the AGAAA motifs was between and overlapped with the two AAAG motifs, and the other motif was downstream and overlapped with the AAAG motif close to the 3′ end of the promoter (**Figure 2A**). Moreover, sequence alignment showed that the cis-regulatory elements of the p184 promoters from Zong31, B73, and Mo17 were identical (**Figure 2B**). To elucidate the element here, as shown in **Figure 2A**, stable transformation of tobacco was performed with five promoter deletion constructs (p184, p160, p155, p151, and p126). GUS activity in the mature pollen grains of the transformed tobacco lines was subsequently measured by histochemical staining assays and fluorometric quantitative analysis. The results showed that p151, lacking one Q-element, had much lower GUS activity in pollen grains than p155, and p155, lacking one AGAAA element, which is a pollen-specific transcription activation cis-regulatory element in the tomato *lat52* gene, had much lower GUS activity than p160. Deletion of the region from –126 to –102 resulted in lacking one Dof core element, destruction of two tandemly repeated Dof core elements and lacking one *lat52* pollen-specific transcription activation cis-regulatory elements (AGAAA). The promoter activity of p160 was much higher than that of p184 indicating that the one Dof core element, especially two tandemly repeated Dof core elements were likely negative regulatory elements (**Figure 2C**). These results implied that a Dof transcription factor might participate in suppressing promoter activity by binding to the AAAG motifs.

### Cloning and Characterization of *ZmDof30*

**FIGURE 3:**
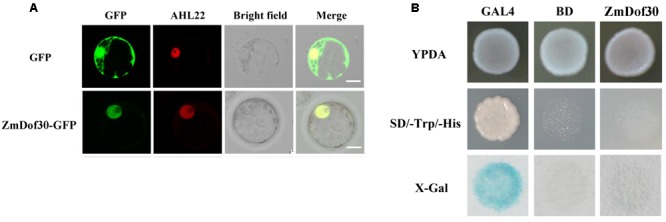
**Subcellular localization and transcriptional activity analysis of Zmdof30. (A)** Subcellular localization of ZmDof30 in maize protoplasts isolated from the second leaf of three-leaf-stage etiolated seedlings. GFP or ZmDof30-GFP was co-transformed into protoplasts with a nuclear marker gene (AHL22-RFP). The scale bar indicates 10 μm. **(B)** ZmDof30 transcriptional activation activity in yeast cells on histidine-free SD medium. GAL4 indicates the positive control and BD indicates the negative control.

The investigation of spatiotemporal expression patterns is useful for understanding gene function. Therefore, we performed expression profiling of 46 maize Dof genes identified by Chen (Chen and Cao, 2015). According to the published tissue RNA-seq data of B73, most Dof family members have low expression levels in pollen, with the exception of *ZmDof1* (GRMZM2G162749) and *ZmDof30* (GRMZM2G178767; Yi et al., 2012). However, *ZmDof1* displayed dramatically higher expression levels in leaves, shoots and whole seedlings, implying that *ZmDof1* may play important roles in leaf development rather than in pollen development. *ZmDof30* was predominantly expressed in whole anthers and pollen (Yi et al., 2012). Therefore, the cDNA sequence of *ZmDof30* was isolated from maize anthers. The ZmDof30 gene contains an ORF of 726bp, encoding a protein with 241 amino acid residues with a conserved zinc finger DNA-binding domain localized between residues 48 to 98. The transcription factor characteristics of ZmDof30 were investigated. A 35S::ZmDof30::GFP plasmid was transfected into maize leaf protoplasts to analyze the subcellular localization of ZmDof30-GFP. AHL22-RFP, a nuclear-localized marker, was co-transfected into maize protoplasts as a positive control. A 35S::GFP plasmid was used as the negative control. ZmDof30-GFP accumulated only in the nuclei of transfected cells, whereas the GFP control was detected in both the nuclei and the cytoplasm (**Figure 3A**). This result confirmed that ZmDof30 localized to the nucleus. A yeast assay system was subsequently used to determine the transcriptional activation activity of ZmDof30 (**Figure 3B**). The coding region of *ZmDof30* was cloned into the pBD-GAL4 vector for fusion with the GAL4 DNA-binding domain. We transformed the fusion plasmid (pBD-ZmDof30) and the control plasmids into the yeast strain YRG-2. The negative control plasmid was the pBD-GAL4 vector with only the GAL4 DNA-binding domain, and the positive control plasmid included both the GAL4 DNA-binding domain and the GAL4 activation domain. The growth of the transfected yeast cells was then examined on nutritionally deficient selection medium (SD/-His). Yeast cells transfected with the positive control construct grew well on histidine-free SD medium and exhibited high β-galactosidase activity. However, no growth was observed for yeast cells transfected with pBD-ZmDof30 or for the negative control, showing that ZmDof30 possessed no transcriptional activation activity in the yeast system and implying that ZmDof30 might act as a transcriptional suppressor.

### ZmDof30 Interacts with the AAAG Motif in p184

**FIGURE 4:**
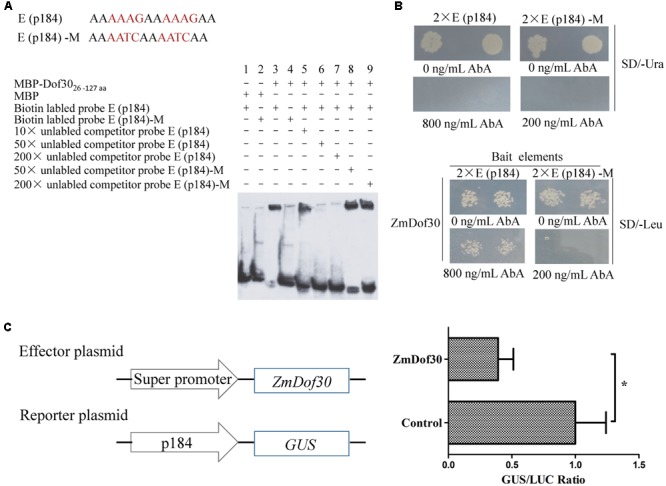
**The binding and regulatory activity of ZmDof30 on the p184 promoter.** Analysis of ZmDof30 DNA-binding specificity with AAAG motifs located in p184 by EMSA **(A)** and a yeast one-hybrid system **(B)**. E (p184) sequence containing two tandem AAAG core motifs; E (p184)-M sequence containing mutations from AAAG to AATC. The oligonucleotide probes with three tandem repeat E (p184) sequence and E (p184)-M sequence were synthesized and labeled with biotin for EMSA. Twin tandem E (p184) and E (p184)-M sequences were cloned into pAbAi vectors as bait. **(C)** Analysis of ZmDof30 regulatory activity on p184 in transient expression assays in tobacco leaves. The p184::GUS reporter plasmid and the pSuper::ZmDof30 effector plasmid were co-transformed into *Nicotiana benthamiana* leaves. The empty vector (pSuper1300) served as a control. GUS activity was normalized to LUC activity derived from an internal control plasmid. The average GUS/LUC value of the control was normalized as 1. The data are represented as the mean ± SD of the results from three independent experiments. Significant differences were assessed by the Student’s *t*-test using a *P*-value < 0.05.

An EMSA was performed to characterize the interaction between ZmDof30 and the Dof core recognition element (AAAG) in p184 *in vitro*. The MBP-ZmDof30_26-127aa_ fusion protein, containing the DNA binding domain of ZmDof30, was expressed in a prokaryotic expression system. The three repeated E (p184) sequence was synthesized in tandem and labeled with biotin. The retardant band indicated that ZmDof30_26-127aa_ could bind to the E (p184) sequence. The binding signal could be reduced by the 50× and 200× unlabeled probe, but not the mutant probes, demonstrating that ZmDof30_26-127aa_ could specifically bind to the E (p184) sequence (**Figure 4A**). We also investigated whether ZmDof30 could bind directly to the E (p184) sequence using a yeast one-hybrid system. The double E (p184) sequence was synthesized in tandem and cloned into pAbAi to construct the bait plasmid and the double E (p184)-M sequence was used to generate the negative bait plasmid (**Figure 4B**). The two bait plasmids were independently transformed into yeast. Subsequently, the minimal concentration of AbA needed for growth inhibition was determined in the yeast cells transformed with bait plasmids. A concentration of 800 ng/mL AbA was chosen for the E (p184) bait yeast strains, and 200 ng/mL AbA was used for the E (p184)-M bait yeast strains (**Figure 4B**). The prey plasmid pGADT7-ZmDof30 was transformed separately into the bait yeast strains. The transformed E (p184) bait yeast cells could grow on medium that lacked leucine but contained 800 ng/mL AbA, while the transformed E (p184)-M bait yeast cells could not grow on medium that lacked leucine and contained 200 ng/mL AbA (**Figure 4B**), indicating that ZmDof30 protein could bind specifically to E (p184) in a yeast system. In conclusion, ZmDof30 could bind to the core recognition element (AAAG) of p184 *in vitro* and *in vivo*.

### ZmDof30 Downregulates p184 Activity in Tobacco

For further analysis, a transcriptional activation experiment was carried out in the leaves of *N. benthamiana*. In this system, the reporter plasmid (p184::GUS), the effector plasmid (pSuper::ZmDof30) and the internal control (35S::LUC) were transformed into *Agrobacterium* (GV3101) individually and then co-infiltrated into leaves of *N. benthamiana*. The negative control was empty pSuper vector. Three days after inoculation, the GUS and LUC activity were assayed in protein extracts prepared from infiltrated leaves. Compared to the control, co-expression of p184::GUS with pSuper::ZmDof30 resulted in a 0.4-fold decrease in relative GUS/LUC activity (**Figure 4C**). The results indicated that ZmDof30 could bind to the E (p184) sequence to suppress promoter activity.

## Discussion

The function of Zm908 in pollen germination has been previously identified (Dong et al., 2013). The overexpression of *Zm908p11* in maize decreased pollen germination efficiency, whereas maize with *Zm908p11* RNAi pollen could produce normal pollen tubes. The success of pollen germination depends on normal or low levels of the Zm908 protein, which can interact with profilin. The appropriate level of Zm908 protein might maintain the dynamic equilibrium between profilin-actin and Zm908p11-profilin-actin, resulting in the normal growth of pollen tubes (Mahoney et al., 1999; Dong et al., 2013). In this study, we aimed to elucidate the regulatory mechanism of *Zm908*. The putative Zm908 promoter was cloned and its activity was analyzed. Tobacco, a heterogeneous experimental system, was widely used to analyze the promoter activity because of its relative high efficiency of transformation, short growth period, and sufficient quantity of pollen grains, Zhou et al. identified several*cis*-regulatory elements involved in the pollen-specific activity of the promoter of the maize gene *SBgLR* using a transgenic tobacco system (Zhou et al., 2010). The cis-regulatory elements directing pistil-specific and pollen-specific expression in the promoter of the *Brassica S* Locus Glycoprotein (*SLG*) gene were also analyzed in tobacco (Dzelzkalns et al., 1993). Here, the activity of the 5′-promoter deletion of pZm908 was analyzed in tobacco, and the result showed that two tandemly repeated Dof core recognition sites (AAAG) in p184 acted as negative elements (**Figure 2C**), indicating that a Dof transcription factor might participate in suppressing the promoter activity.

Members of the *Dof* gene family are clearly vital for plant development in a number of cases. *PBF* (prolamin-box binding factor) and *ZmDof3*, both of which are members of the Dof transcription factor family in maize, were reported to play critical roles in seed development (Marzabal et al., 2008; Qi et al., 2016). *PBF* is predominantly expressed in embryos, endosperm and seeds, and it positively regulates expression of the *zein* gene in developing maize seed (Marzabal et al., 2008). *ZmDof3*, another endosperm-specific Dof gene, has been implicated in aleurone development and starch accumulation in maize endosperm (Qi et al., 2016). *Dof1*, which was first cloned from maize by Yanagisawa (Yanagisawa and Sheen, 1998), is an example of a Dof transcription factor that acts on plant pollen development. It is expressed in pollen and represses the pollen-specific *Zm401* gene to control pollen development (Chen et al., 2012). According to previous RNA-seq data from different tissues in B73, *ZmDof30* has relatively higher expression levels in pollen than other ZmDofs and is predominantly expressed in whole anthers and pollen (Yi et al., 2012). ZmDof30 was successfully cloned in this study. The subcellular localization analysis showed that ZmDof30-GFP accumulated only in the nucleus of maize protoplasts (**Figure 3A**). And ZmDof30 possessed no transcriptional activation activity in a yeast system (**Figure 3B**). The EMSA and yeast one-hybrid results indicated that ZmDof30 interacted directly with p184 by binding to the AAAG element (**Figures 4A,B**). Agroinfiltration of *N. benthamiana* leaves is a widely used transient system to analyze the transcriptional regulation of the target genes. For example, a transient transcription assay in leaves of *N. benthamiana* revealed that the 27-kD *γ-zein* promoter was transactivated by OHPs and PBF (Zhang et al., 2015). In our study, the regulation of ZmDof30 on p184 were analyzed in the leaves of *N. benthamiana* and the results showed that ZmDof30 could suppress the promoter activity of p184 (**Figure 4C**). All of these results indicate that ZmDof30 is a Dof transcription factor and functions as a Zm908 transcriptional repressor, providing evidence that a Dof transcription factor participates in pollen germination. The expression profile of a gene may shed light on its biological function. In addition to pollen, *ZmDof30* was also predominantly expressed in whole anthers, implying that *ZmDof30* may also be associated with the maize pollen development processes.

The binding of Dof transcription factors mediates the activation or suppression of gene transcription, depending on the target gene. One Dof gene, *DAG1* (Dof affecting germination), affected seed germination when expressed in the maternal testa layer of the seed. The *DAG1* mutant line showed reduced dormancy (Papi et al., 2000). However, the opposite effect was observed in the *DAG2* mutant (Gualberti et al., 2002). It seems that these two gene paralogs acted antagonistically on the same set of target promoters. Through analysis of 35S-AtDOF4:2 and RNAi-AtDOF4:2 plants, it was revealed that *AtDOF4:2* was involved in phenylpropanoid metabolism in *A. thaliana*, negatively influenced flavonoid synthesis, and positively affected hydrocinnamic acid production. The variable terminal region of Dof had transcriptional regulation activities, mediating activation or repression of gene expression by interacting with other regulatory proteins, including the Dof domain itself (Yanagisawa and Schmidt, 1999; Yanagisawa, 2000), a bZIP transcription factor (Cao et al., 1997), a Myb transcription factor (Vicente-Carbajosa et al., 1997; Diaz et al., 2002) and other regulatory proteins. Dof1 enhanced the transcriptional activity of the C4 phosphoenolpyruvate carboxylase promoter in leaves (Yanagisawa and Sheen, 1998; Yanagisawa, 2000). Furthermore, a transcriptional activation domain was identified in the C-terminus of Dof1 (Yanagisawa, 2001). However, Dof1 repressed the expression of the pollen-specific gene *Zm401*, thereby controlling pollen development and suggesting that there may be a suppressor that interacts with the Dof1 protein to abolish its transcription-activating activity (Chen et al., 2012). In this study, ZmDof30 possessed no transcription activation activity in a yeast system and suppressed the promoter activity of *Zm908* in transient expression assays in *N. benthamiana*. These results imply that ZmDof30 might mediate the repression of Dof1 transcriptional activity by interacting with Dof1. However, further analyses are needed.

## Conclusion

The *Zm908* promoter was cloned and identified. pZm908 is pollen-specific, and the –126 to –102 region acts as a negative element. ZmDof30 could bind to the AAAG elements of p184 and negatively regulate *pZm908* activity in tobacco leaves. ZmDof30 may function as a transcriptional repressor of *Zm908* in pollen. These results provide a better understanding of the regulation of the *Zm908* gene and the pollen-specific *Zm908* promoter may have a potential for application in genetically engineering male sterility.

## Author Contributions

JY and JP conceived and designed the research. JP prepared materials, designed and performed the research, and wrote the original manuscript. XQ, XC, and NL participated in the preparing materials. JY revised thoroughly the manuscript and finalized the manuscript. All authors read and approved the final manuscript.

## Conflict of Interest Statement

The authors declare that the research was conducted in the absence of any commercial or financial relationships that could be construed as a potential conflict of interest.
