# Supplementary material for: ZmDof30 Negatively Regulates the Promoter Activity of the Pollen-Specific Gene Zm908
Source: Front Plant Sci. 2017 May 1;8:685. doi: 10.3389/fpls.2017.00685 (PMC5410603; doi:10.3389/fpls.2017.00685)
Supplement: Supplementary file 1 [file Table_1.docx]

Table S1 Primers and the enzyme digest sites used in construction of GUS reporter plasmids for truncation series of pZm908.

|  | The length of |  |  |
| --- | --- | --- | --- |
| Primer name | the promoter segment (bp) | Primer sequence (5'-3') | Restriction site |
| p908-GUS-F | 2126 | cgcGCATGCGGTGTGCGTAATGACTC | *Sph*I |
| p1670-GUS-F | 1670 | cgcGCATGCGCTTACTAAGATTGGATC | *Sph*I |
| p1126-GUS-F | 1126 | cgcGCATGCAACCGCCCGATCTCGTC | *Sph*I |
| p789-GUS-F | 789 | cgcGCATGCAAAAGGGGTAACGCAC | *Sph*I |
| p586-GUS-F | 586 | cgcGCATGCCACAATAGAAACATACT | *Sph*I |
| p244-GUS-F | 244 | cgcGCATGCAGAAGTCTAGCAAAATTG | *Sph*I |
| p184-GUS-F | 184 | cccAAGCTTCCTGAGCTGACAGTAA | *Hin*dIII |
| p160-GUS-F | 160 | cccAAGCTTAGAAAAAATGATGAAT | *Hin*dIII |
| p155-GUS-F | 155 | cccAAGCTTAAATGATGAATAGTGT | *Hin*dIII |
| p151-GUS-F | 151 | cccAAGCTTGATGAATAGTGTTGGG | *Hin*dIII |
| p126-GUS-F | 126 | cccAAGCTTGCCCAAGTCGGCCCGCAG | *Hin*dIII |
| p908-R |  | cgcGGATCCGAAACATATGAATAGTAC | *Bam*HI |
